# Peer tutoring in clinical communication teaching: the experience of 1st year students and their peer tutors

**DOI:** 10.15694/mep.2018.0000135.1

**Published:** 2018-06-20

**Authors:** Athina Belsi, Ged M. Murtagh

**Affiliations:** 1 Imperial College London

**Keywords:** Clinical Communication, Peer Tutoring, Feedback, Facilitation, Teaching Experience

## Abstract

This article was migrated. The article was marked as recommended.

This study explored the impact of peer tutoring in Clinical Communication teaching as it was expressed in the views of Year 1 students and their Peer Tutors at Imperial College London.

**Methods:** a mixed methods approach was used combining questionnaires and focus groups. Quantitative findings were analysed using SPSS v23 and qualitative findings were analysed using Framework Methodology.

**Results:** the findings suggest a very positive experience for both Year 1 students and their Peer Tutors with the former reporting feeling supported to practice and improve on their Clinical Communication Skills in a collaborative environment, being taught and learning from peers who would share their past educational experiences. No significant differences were found between students taught by Peer Tutors and those taught by Course Tutors. Peer Tutors on the other hand, reported an equally positive experience, which gave them an insight into teaching, improved their leadership and feedback skills, enhanced their confidence and helped them reflect on their own Clinical Communication skills.

**Conclusion:** peer tutoring has many advantages as an educational method in medical education and Clinical Communication teaching and should be promoted in medical curricula.

## Introduction

Peer tutoring (also known as peer assisted learning) has been described as ‘the development of knowledge and skill through active help and support among status equals or matched companions ( Topping, 2005) and may refer to the experience of being a peer tutor as well as being taught by a peer tutor. Peer tutoring (PT) is an approach where people from similar social groupings but not professional teachers themselves, help each other to learn, while learning themselves. In higher education, PT has been an area of growing interest ( Boud, Cohen, & Sampson, 2001) and is now a well-established educational method used in undergraduate and postgraduate studies ( Ten Cate & Durning, 2007b; Topping, 1996). In the field of medical and allied health education PT has been adopted by many undergraduate medical schools globally ( Carr et al., 2016; Nestel & Kidd, 2003; Yu et al., 2011) and can occur formally as a component of the official curriculum ( Goodfellow & Schoefield, 2001; Nestel & Kidd, 2003; Rudy, Fejfar, Griffith, & Wilson, 2001) as well as informally, for example when students help each other in preparation for exams. The importance of PT for the medical profession has been further highlighted in the UK by the General Medical Council, which has characterised the ability to effectively teach, reflect and learn as a prerequisite for new doctors (GMC, 2015).

Studies suggest that peer tutoring in health education can be beneficial for both tutees and peer tutors (PTs), as well as for institutions ( Pasquale & Pugnaire, 2002; Ten Cate & Durning, 2007b). A range of benefits have been identified, including economic advantages at institutional level, as peer tutoring is considered a teaching resource-saving measure, especially considering the increasing number of medical students. Other benefits include the promotion of a collegial behaviour and cognitive development of students: PT can create a safe and comfortable learning environment, where peers are supportive of each other and mistakes can be corrected and used as a learning tool ( Lockspeiser, O’Sullivan, Teherami, & Muller, 2006; Secomb, 2008). Similarly, Bulte et al (Bulte, Betts, Garner, & Durning, 2007) discussed the positive effect of cognitive congruence between peer tutors and tutees: tutees feel their tutors cognitively close to themselves and thus may feel more at ease to express questions and concerns. This proximity, not only cognitively, but also socially, has also been found to help towards the formation of collegial attitudes ( Nestel & Kidd, 2003): being all students, peer tutors and tutees work in a collaborative and co-operative environment ( Bruffee, 1999). Indeed, in a study of the impact of peer tutoring on first year medical students, Nestel & Kidd (2003) found that students can learn from supportive interactions with- and the experiences of senior peers who, having been in the past exposed in the same educational content and activity as the latter, can share their experiences and learning journey with their younger colleagues.

However, peer tutoring has been associated with positive educational outcomes not only for the tutees, but also for Peer Tutors: while the former have reported increased student satisfaction and enriched learning experience, the latter have also reported training in and an improvement in leadership and teaching skills as well as enhanced confidence ( Burgess, McGregor, & Mellis, 2014; Ten Cate & Durning, 2007a). Peer tutors have also been found to gain valuable experience in facilitation and communication skills ( Nestel & Kidd, 2005), which are important skills for medical professionals.

### Clinical Communication and peer tutoring

On the Clinical Communication (CC) programme we run at Imperial College London, peer tutoring is an important component of the Year 1 simulated patient encounters, where Year 1 students are taught either by a Course Tutor or by a pair of Peer Tutors; these are Year 3 students who volunteer to participate to the programme. For the purposes of these sessions, Year 1 students attend in groups of three and each student has an opportunity to interview a simulated patient whilst being observed by their peers and the Tutor (Course- or Peer-). Following the interview, the student receives feedback on their performance from their peers, the simulated patient and the Tutors. The aim of this paper is to evaluate the student experience of the Peer Tutor programme in Clinical Communication by comparing the views of students taught by Peer Tutors to those taught by Course Tutors. A secondary aim is to explore the views of the Peer Tutors on their teaching experience with a view to inform current practice.

## Method

All year 1 students attending the simulated patient interviews in the academic year 2016-17 were invited to participate in a survey (N=329). Equally, all Year 3 students who volunteered to act as Peer Tutors, and subsequently facilitated sessions, were invited to participate in the evaluation survey (N=21). In total, there were 57 groups of Year 1 students, 34 of which were taught by Course Tutors and 23 by Peer Tutors. Year 1 students were randomly allocated to sessions and were taught either by a Course- or a Peer Tutor, while the latter selected the sessions they would facilitate based on their availability and other course commitments. This study was a service evaluation and therefore was exempt from formal ethics approval processes.

### Peer Tutor Recruitment and training

All year 3 students were invited to participate in the Peer Tutor programme at the end of a Clinical Communication lecture given in year 3. By volunteering, participating students committed to attend a training workshop and to facilitate two sessions each. The training consists of a 3-hour long workshop which takes place a week before the Peer Tutor sessions were due to start; It is designed to familiarise the Peer Tutors with the aims and content of the simulated patient teaching as well as the relevant educational theory underpinning the teaching. After attending a short presentation, the prospective Peer Tutors were given a demonstration of how a simulated patient session was run. In the latter part of the workshop they facilitated a session themselves with the aid of course tutors. At the end, the Peer Tutors selected the sessions they would facilitate and were given a peer tutor guide describing the structure and content of the session as well as relevant educational material.

### First Year Students: questionnaires

All first-year students attending their simulated patient interview were asked to fill in a questionnaire at the end of their session. The questionnaire comprised of 7 statements that students rated using a five-point Likert scale, ranging from ‘strongly agree’ to ‘strongly disagree’. The statements explored students’ ratings on their level of confidence about talking to a patient; whether their learning objectives from the session were achieved; understanding their individual clinical communication strengths and weakness; and finally, on receiving good constructive feedback and feeling motivated to improve their clinical communication skills. There was also space for free-text comments.

### Peer Tutors: questionnaires and focus groups

All Peer Tutors participating in the programme were asked to fill in a questionnaire at the end of the second session they facilitated so that they would have had the full experience of peer tutoring on the clinical communication course. The questionnaire comprised of five statements and three open-ended questions. The statements, which the Peer Tutors rated using a five-point Likert scale, explored their views on the training session they received; whether being a PT helped them reflect on their own CC skills; if their leadership skills have improved and how they rated their experience as PTs; the three open questions explored the Peer Tutors’ self-assessment of their role and any challenges they faced.

Quantitative data from the questionnaires were analysed using SPSS v23. For Year 1 students’ responses chi-square and Fisher’s exact tests were performed to compare the responses of students being taught by Course Tutors and those taught by Peer Tutors. Descriptive analysis was performed for the questionnaires collected from the Peer Tutors.

Free text responses were analysed using Framework Methodology, a method which classifies and organises data into key themes, concepts and emergent categories, which can then be synthesised and refined ( Ritchie & Lewis, 2003).

## Results

### Year 1 students

A total of 275 Year 1 students completed a questionnaire at the end of their simulated patient session thus giving a response rate of 84%.

From the 275 Year 1 students, 128 were taught by Course Tutors and 147 were taught by Peer Tutors. Table 1 shows how Year 1 students evaluated their experience of the simulated patient session in terms of their levels of confidence about talking to a patient before and after the session; whether their learning objectives were achieved; if their tutor helped them understand their strengths and weaknesses in patient-centred interviewing or gave good constructive feedback; and whether they felt motivated to improve their clinical communication skills. For analysis purposes, the five options were collapsed into three: ‘agree’, ‘neutral’ & ‘disagree’. No statistically significant differences were found between the two groups taught by course tutors or by PTs in any of the seven aforementioned areas.

Notably, only the Year 1 students taught by Peer Tutors offered free text comments. The comments were mainly around three thematic areas which attracted similar number of responses: the *friendly and supportive environment encountered in the sessions;* the *very detailed feedback the students felt they received* and *how they related to the peer tutors’ previous experiences of CC within the course but also in the clinical environment.*

Regarding the *friendly and supportive environment* encountered in the sessions, students commented that this helped make the interview process more enjoyable, despite being recorded and watched by their peers. In addition, the very specific and detailed feedback was also mentioned as this was found to help them identify their strengths and weaknesses.

*“the tutors were very friendly and made the whole interview experience seem enjoyable. They gave very positive feedback, but also constructive feedback”* (Y1, 157)

Similarly, it was reported that the encouraging manner in the way the Peer Tutors gave their feedback helped students to reflect on their skills and improve.

*“really well structured feedback, with constructive criticism that was easy to understand and they suggested useful easy to improve you next time”* (Y1, 178)

Another area frequently mentioned was how the Peer Tutors shared their own experiences in Clinical Communication both when they were attending the course when they were in Year 1, but more importantly when they started applying the knowledge and skills they had acquired in the clinical setting. Sharing their experiences as well as giving useful tips gave Year 1 students a ‘ *clearer perspective in clinical communication*’, but also helped them to consider the wider implications of communicating with patients.

*“.. they also give great tips, probably things that you can’t learn without experience’* (Y1, 155)

*“made me really consider all aspects of communication, allowed me to consider more aspects than I would have”* (Y1, 175)

*“speaking with experiences, gave me a clearer perspective on CC”* (Y1, 176)

### Peer Tutors

In total 17 out of the 21 PTs participating in the programme completed the questionnaire, giving a response rate of 81%. Again, the five categories were collapsed into three: ‘agree’, ‘neutral’ and ‘disagree’. Their responses in the first four statements they were asked to rate gravitated towards the ‘agree’ option. These were:

*I found the training session useful in preparation for my role as a Peer Tutor*

*I felt comfortable acting as a Peer Tutor*

*Being a Peer Tutor helped me in my personal reflection of my clinical communication skills*

*I feel my leadership skills have improved as a result of being a peer tutor*

As for the last statement ‘ *I would rate my experience as Peer Tutor as:*’ eleven students (64.7%) responded it was ‘excellent’ and six students (35.3%) rated it as ‘very good’.

There were also three free text questions where Peer Tutors were asked to record their thoughts on what went well and what they could have done better as Peer Tutors, as well as on the challenges they faced. Thematic analysis of their responses suggested that on the whole, students believed they best performed by sharing their own experiences with the younger students. Referring to their own practice of CC and discussing the different contexts they communicated with patients was thought to help Year 1 students feel comfortable and reassured, especially in case they were concerned about their communication skills. It was also suggested that incorporating in the feedback relevant and interesting examples from their own practice, examples that Year 1 students could relate to, seemed to improve the confidence of the latter.

*“share my experience and knowledge with the Y1 students”* (PT4)

*“gave good feedback and helped Y1 students be more confident”* (PT7)

Nevertheless, using appropriate examples in the feedback process was an area some Peer Tutors felt they wanted to improve on. Similarly, ensuring they had addressed and met the learning goals of their tutees was another element of their role they would like to develop further.

*“make sure that all their learning goals were addressed and discussed”* (PT9)

*“point out few improvements and be more specific with some parts of feedback”* (PT16)

As was running the sessions in a timely manner and follow a structure to it.

Finally, Peer Tutors referred to some of the challenges they faced; these included managing time pressures and ensuring all elements of the sessions had been addressed; giving constructive feedback, without sounding too harsh on Year 1 students and dealing with students who thought they couldn’t improve their CC skills. One Peer Tutor commented:

*“time pressure-I had to stick by it without rushing and dropping the quality of the feedback..”* (PT1)

while another one identified as the major challenge the need to balance feedback:

*“giving the right amount of things to improve, because I wanted to give them loads of tips and talk about everything, but also didn’t want to overload them so getting the balance right was a bit difficult”* (PT2)

## Discussion

This study sought to explore the views of students who participated in a peer tutoring programme in both ends of the teaching spectrum: either as Peer Tutors or as students. Responses from both groups suggest an overall very positive experience from the programme, in line with previous research in the same institution and elsewhere ( Burgess et al., 2014; Nestel & Kidd, 2003, 2005; Yu et al., 2011) thus reinforcing peer tutoring’s important role as a teaching method in medical education.

The response rate for Year 1 students in this study was higher in those groups taught by Peer Tutors as opposed to the groups taught by course tutors (n=147 cf n=128). Whilst the exact reason for this is not known, it could be speculated that Peer Tutors were more diligent in administering the questionnaires at the end of the teaching sessions. Equally, only Year 1 students taught by Peer Tutors gave answers to the free text questions, rather than students taught by Course Tutors. Nevertheless, the overall response rate of 84% suggested that the findings could be considered representative for this year of students.

Interestingly, the findings revealed no statistically significant differences in the views of students taught in the two groups, in any of the seven statements they were asked to rank. This is an noteworthy finding as it contrasts to what Nestel and Kidd (2003) had found in a similar study at the same institution, where more students taught by Course Tutors had reported meeting their learning objectives when compared to students taught by Peer Tutors. Another main difference lies in the fact that in our study Year 1 students taught by Peer Tutors commented on the very detailed feedback they received during the session; this again contrasts to the findings of the Nestel and Kidd study (2003), where students taught by Course Tutors reported receiving more detailed feedback. Given that the preparatory workshop that was offered to our Peer Tutors was similar to that offered by Nestel and Kidd. one cannot be sure for the reason for this difference in responses. A possible explanation for our findings could be that the principles of feedback that were used for the training were the same that have been used throughout the CC sessions and hence the Peer Tutors students had already been exposed to this type of feedback (receiving and giving) in their first two years of studies.

Year 1 students also commented on the very supportive environment they encountered during the sessions. While this is a positive finding for both groups of Tutors, it reinforces a benefit of peer tutoring in that it can create a warm and inclusive environment which fosters affective support ( Dornan et al., 2014); such a learning environment can, in turn, promote supportive interactions between students and Tutors. Indeed the concept of ‘social congruence’ which is found in peer tutoring ( Lockspeiser et al., 2006), as students and tutors have similar social roles ( Burgess, Dornan, Clarke, Menezes, & Mellis, 2016), can create a safe environment where mistakes can be corrected in a collaborative and cooperative manner. The same finding further suggests that peer tutoring could promote positive collegial behaviours, which are critical for future positive working relationships.

Furthermore, Year 1 students commented on how they were able to relate to the Peer Tutors’ educational experiences and take advantage of useful tips the latter shared with them. This is another notable finding as it highlights the value of the ‘cognitive congruence’ ( Cornwall, 1980; Yu et al., 2011) that is present in peer tutoring; Year 1students perhaps felt more at ease to discuss the challenges they faced in developing the CC skills with the Peer Tutors as they felt them closer to their ‘fund of knowledge’ ( Ten Cate & Durning, 2007b) and thus able to identify with their uncertainties, discuss similar educational experiences and explain concepts appropriately.

On the other hand, the Peer Tutors in our study also feedback positively on the opportunity they had to share their own experiences with younger students, while at the same time reflect on their own teaching practice as well as on their CC skills. This is an interesting finding for two reasons: first it suggests that teaching another student-using existing knowledge and skills-can potentially encourage a deeper engagement with learning ( Burgess et al., 2014). Secondly, the finding strengthens the importance of peer teaching in promoting self-awareness of skills, including teaching skills, but also in this study, Peer Tutors’ clinical communication skills.

In addition, the Peer Tutors noted that by acting as teachers they could practise their facilitation as well as feedback skills but also felt the responsibility of running a teaching session, including facing challenges like time keeping and tailoring their feedback to the recipient. Previous research has suggested that practising the above skills can promote student leadership and improve confidence ( Secomb, 2008), as well as help Peer Tutors better articulate their ideas and emotions, transitioning from student to teacher mode ( Burgess et al., 2014). in addition, it has been suggested that exposure to teaching skills and principles should start in medical school and continue through to medical practice in a sequential manner, so that medical graduates can develop the necessary competencies and attitudes to become competent teachers ( Dandavino, Snell, & Wiseman, 2007; General Medical Council, 2016).

A few limitations of the current study should be acknowledged. While all Year 1 students were invited to participate to the study, the Peer Tutors students were a self-selected group and as such they may hold certain positive attitudes towards teaching and facilitating sessions. In addition, the sample size of the Peer Tutors was rather small as the CC programme can accommodate a certain number of them. Finally, the findings come from one academic year only, further research would be useful in replicating our results.

## Conclusions

The purpose of this study was to explore the role of peer tutoring in Clinical Communication teaching as this was perceived both by students and Peer Tutors, using a mixed methodology. The findings suggest overall a very positive experience and are consistent with past research on the multi-level benefits of peer tutoring in medical education. Even more so, the findings suggest that peer tutoring can be equally effective as a teaching method in CC skills training in the first year of medical school. As the ability to teach and reflect on their own practice is one of the requirements for future medical professionals, further research should examine how peer tutoring can be promoted within the medical curricula easing the transition from ‘teacher’ medical student to a ‘teacher’ doctor.

## Take Home Messages

• Peer tutoring can be effectively used in clinical communication teaching
• Year 3 students acting as Peer Tutors reported gaining valuable experience in teaching, leadership, facilitation skills as well as gaining a deeper insight into their own clinical communication skills
• Year 1 students enjoyed being taught by peers who shared past experiences
• Peer tutoring can contribute to the transition from medical student to medical teacher

## Notes On Contributors

Athina Belsi is a Senior Teaching Fellow in the Department of Surgery and Cancer. She is a social scientist and Course Leader for the Clinical Communication Programme. Previously she has held teaching and research posts at the Department of Primary Care and Public Health at Imperial College and at King’s College London.

Ged Murtagh is a Senior Lecturer in Communication in the Department of Surgery and Cancer. He is a social scientist by training and Course Leader for the Clinical Communication Programme. Previously he has held teaching and research positions at the Universities of Surrey and Leicester.

## Appendices

**Table 1.** First-year students’ views on their experience of their simulated patient interviews with medical teachers or Peer Tutors (N=275)

	Agree		Neutral		Disagree	
	Course Tutor N (%)	Peer Tutor N (%)	Course Tutor N (%)	Peer Tutor N (%)	Course Tutor N (%)	Peer Tutor N (%)
I felt comfortable about talking to a patient before the session	76 (59.4)	99 (67.3)	36 (28.1)	33 (22.4)	16 (12.5)	15 (10.2)
I felt more comfortable about talking to a patient after the session	120 (93.8)	144 (98)	4 (3.1)	2 (1.4)	4 (3.1)	1 (0.7)
My learning objectives were achieved at the session	115 (89.8)	138 (93.9)	12 (9.4)	7(4.8)	1 (0.8)	2 (1.4)
The tutor helped me understand my strengths in patient-centred interviewing	128 (100)	145 (53.1)	0	2 (1.4)	0	0
The tutor helped me understand my weaknesses in patient-centred interviewing	127 (99.2)	145 (98.6)	1 (1.4)	2 (1.4)	0	0
The tutor gave good constructive feedback	128 (100)	146 (99.3)	0	1 (0.7)	0	0
I feel motivated to improve my clinical communication skills	126 (98.4)	146 (99.3)	2 (1.6)	1 (0.7)	0	0
