# Supplementary material for: Peer tutoring in clinical communication teaching: the experience of 1st year students and their peer tutors
Source: MedEdPublish (2016). 2018 Jun 20;7:135. [Version 1] doi: 10.15694/mep.2018.0000135.1 (PMC10699383; doi:10.15694/mep.2018.0000135.1)
Supplement: Peer review history

# Reviewer report: Reviewer response for version 1

**DOI:** 10.21956/mep.19603.r29118

**Published:** 2018-07-06

**Authors:** Sateesh Babu Arja

**Affiliations:** 1 Avalon University School of Medicine

This review has been migrated. The reviewer awarded 4 stars out of 5 I enjoyed reading this paper. The authors clearly explained the importance of having peer tutors in medical education. Authors clearly detailed the research methods and results and it is a well-written paper. Peer tutoring encourages collaborative work and reduces competition which can foster team values. This is a very important trait or attributes that we need to develop among healthcare professionals as this healthcare professionals are interdependent and work in teams once they enter into practice. Currently, we also have peer tutoring programs at our university. We also offer incentives for the senior students who are involved in peer tutoring. We are using peer tutoring for biomedical sciences but not for clinical skills or communication skills. It is good to know that peer tutoring can work out even for teaching communication skills. This article is very useful for the teachers involved in teaching communication skills.

Reviewer Expertise:

NA

No decision status is available

# Reviewer report: Reviewer response for version 1

**DOI:** 10.21956/mep.19603.r29116

**Published:** 2018-06-21

**Authors:** Ronald M Harden

**Affiliations:** 1 AMEE

This review has been migrated. The reviewer awarded 4 stars out of 5 A paper well worth reading. While the value of peer teaching is increasingly recognised, some teachers are still reluctant to adopt the approach believing that teachers are there to teach and students are there to learn. It is valuable to see additional studies such as this one that demonstrate clear advantages of the approach both for the student tutor and the tutee. The training of the tutors is important and it is of interest to see how this was achieved. The comparison of the students attitudes and evaluation of the student and the staff tutors is well described. Perhaps a follow-up study could examine how the different groups performed when their competencies were assessed eg in an OSCE.

Reviewer Expertise:

NA

No decision status is available

# Reviewer report: Reviewer response for version 1

**DOI:** 10.21956/mep.19603.r29117

**Published:** 2018-06-20

**Authors:** P Ravi Shankar

**Affiliations:** 1 American International Medical University

This review has been migrated. The reviewer awarded 4 stars out of 5 I enjoyed reading this manuscript about peer tutoring in a medical school in the United Kingdom. The article is well-written and the methods are clearly described. The authors have described the organization of the sessions in detail. A heavy academic schedule and finding time in the curriculum for peer teaching is a challenge and the authors have described clearly how they overcome the challenge. This article will be of general interest to all medical educators.

Reviewer Expertise:

NA

No decision status is available
